# Behavior of lung ultrasound findings during spontaneous breathing trial

**DOI:** 10.5935/0103-507X.20170038

**Published:** 2017

**Authors:** Ana Carolina Peçanha Antonio, Cassiano Teixeira, Priscylla Souza Castro, Augusto Savi, Juçara Gasparetto Maccari, Roselaine Pinheiro Oliveira, Marli Maria Knorst

**Affiliations:** 1 Adult Intensive Care Unit, Hospital Moinhos de Vento - Porto Alegre (RS), Brazil.; 2 Adult Intensive Care Unit, Hospital Mãe de Deus - Porto Alegre (RS), Brazil.; 3 Adult Intensive Care Unit, Hospital de Clínicas de Porto Alegre - Porto Alegre (RS), Brazil.; 4 Postgraduate Program in Pneumology, Universidade Federal do Rio Grande do Sul - Porto Alegre (RS), Brazil.

**Keywords:** Ventilator weaning, Respiration, artificial, Ultrasonography, Respiratory failure, Pulmonary edema

## Abstract

**Objective:**

We aimed to investigate a potential association between B-lines and weaning failure.

**Methods:**

Fifty-seven subjects eligible for ventilation liberation were enrolled. Patients with tracheostomy were excluded. Lung ultrasound assessments of six thoracic zones were performed immediately before and at the exnd of the spontaneous breathing trial. B-predominance was defined as any profile with anterior bilateral B-pattern. Patients were followed up to 48 hours after extubation.

**Results:**

Thirty-eight individuals were successfully extubated; 11 failed the spontaneous breathing trial and 8 needed reintubation within 48 hours of extubation. At the beginning of the T-piece trial, B-pattern or consolidation was already found at the lower and posterior lung regions in more than half of the individuals and remained non-aerated at the end of the trial. A trend toward loss of lung aeration during spontaneous breathing trials was observed only in the spontaneous breathing trial-failure group (p = 0.07), and there was higher B-predominance at the end of the trial (p = 0.01).

**Conclusion:**

A loss of lung aeration during the spontaneous breathing trial in non-dependent lung zones was demonstrated in subjects who failed to wean.

## Abstract (pt)

**Objetivo:**

Investigar potencial associação entre a presença de linhas B e a falha do desmame.

**Métodos:**

Foram inscritos 57 pacientes elegíveis para liberação da ventilação. Excluíram-se pacientes com traqueostomia. Realizou-se avaliação ultrassonográfica pulmonar de seis zonas torácicas imediatamente antes e após o final da tentativa de respiração espontânea. Definiu-se a predominância de linhas B como qualquer perfil com padrão B bilateral anterior. Os pacientes foram seguidos por 48 horas após a extubação.

**Resultados:**

Foram extubados com sucesso 38 pacientes; 11 tiveram falha da tentativa de respiração espontânea; e 8 necessitaram de reintubação dentro de 48 horas após extubados. No início da tentativa com peça T, já se observava padrão B ou consolidação nas regiões posterior e inferior dos pulmões em mais de metade dos indivíduos, que permaneceram não aeradas ao final da tentativa. Observou-se certa tendência à perda da aeração pulmonar durante a tentativa de respiração espontânea apenas no grupo com falha da tentativa de respiração espontânea (p = 0,07), assim como maior predominância de padrão B ao final da tentativa (p = 0,01).

**Conclusão:**

A perda de aeração pulmonar durante a tentativa de respiração espontânea em áreas pulmonares não dependentes foi demonstrada em pacientes que tiveram falha do desmame.

## INTRODUCTION

The weaning process comprises progressive withdrawal from invasive ventilatory support until removal of the endotracheal tube and might represent approximately 42% of the duration of mechanical ventilation (MV).^(1-3)^ In the intensive care unit (ICU), the use of multiple respiratory indices to dictate the weaning process have been largely supplanted by the more rapid and predictive spontaneous breathing trial (SBT).^(4-6)^ Better assessments of patients before and during an SBT are of paramount importance to predict weaning failure and to focus treatment that could reduce the time spent on artificial ventilation.

Cardiac dysfunction is a well-established cause of weaning failure, representing 40% of all weaning failures.^(7,8)^ Switching a patient from positive pressure ventilation to spontaneous breathing reinstitutes negative inspiratory intra-thoracic pressure, thus increasing venous return, central blood volume, and left ventricular afterload. This normal condition, often an effort test for the patient, could decompensate cardiorespiratory function in cases of volume overload and left ventricular systolic or diastolic dysfunction.^(9)^ Spontaneous breathing trial-induced increases in extravascular lung water (EVLW) and B-type natriuretic peptide are reliable alternatives to the pulmonary artery catheter for diagnosing weaning-induced pulmonary edema.^(10)^

Lung ultrasound (LUS) is a basic application of critical ultrasound - a loop associating urgent diagnoses with immediate therapeutic decisions.^(11,12)^ Multiple B-lines are considered the sonographic sign of lung interstitial syndrome.^(11-13)^ The so-called B-pattern has been validated to measure EVLW,^(14-16)^ and emergency presentations with shortness of breath, patients with known heart failure and fluid overload in the context of chronic hemodialysis have all been studied with LUS.^(17)^ LUS has a demonstrated sensitivity of 97-100% and specificity of 95% for detecting acute pulmonary edema.^(13,18)^ In patients with suspected interstitial syndrome, a negative lung ultrasound examination is superior to conventional chest radiography in ruling out significant interstitial syndrome.^(12)^

Reasons for failure to wean from MV support are often multifactorial and involve a complex interplay between cardiac and pulmonary dysfunction. A recent review suggests the intensivist might productively use ultrasonography to identify impediments to successful extubation.^(19)^ To further investigate the relationship between B-lines and MV-weaning, we report the LUS findings of 57 MV subjects subjected to SBT, immediately before and after the procedure.

## METHODS

Nonconsecutive individuals older than 18 years of age who had undergone invasive MV for at least 24 hours were enrolled from a medical-surgical, semi-closed unit in a private hospital that is covered full-time by intensivists. Individuals with a tracheostomy were excluded. The Research Ethics board approved the study and waived the requirement for informed consent. The study is registered as NCT02022839 at ClinicalTrials.gov.

Patients were assessed daily for eligibility for weaning according to improvement of underlying condition that led to acute respiratory failure; alert and able to communicate; adequate gas exchange, as indicated by an arterial pressure of oxygen of at least 60mmHg with an inspired fraction of oxygen < 0.40; no significant respiratory acidosis; rapid shallow breathing index equal to or less than 105 cycles per minute per liter; and vasoactive drugs at low and stable doses (norepinephrine doses lower than 0.12µg per kilogram per minute or dopamine equivalent doses).

Spontaneous breathing trial failure was defined as inability to tolerate a T-piece trial of spontaneous breathing for 30 to 120 minutes, in which case subjects were not extubated. The breathing trial was interrupted if the patient developed signs of respiratory discomfort (respiratory frequency > 35 breaths per minute, arterial oxyhemoglobin saturation < 90%, use of accessory respiratory muscles or paradoxical thoracoabdominal ventilation), tachycardia (heart rate more than 140 beats per minute), hemodynamic instability (systolic blood pressure less than 90mmHg or 20% over basal levels) or change in mental status (drowsiness, coma, anxiety). Extubation failure was defined as the need for reintubation within 48 hours after planned removal of the artificial airway.

Demographic data including age, sex, race, comorbidities, and severity of illness at the time of ICU admission, reason for the initiation of MV, physiological weaning predictors and fluid balance in the 48 hours preceding SBT were recorded. The presence of diastolic or systolic left ventricular dysfunction (the latter condition defined as ejection fraction < 45%) was documented according to formal echocardiogram report dated up to six months prior to admission.

**Figure 1:**
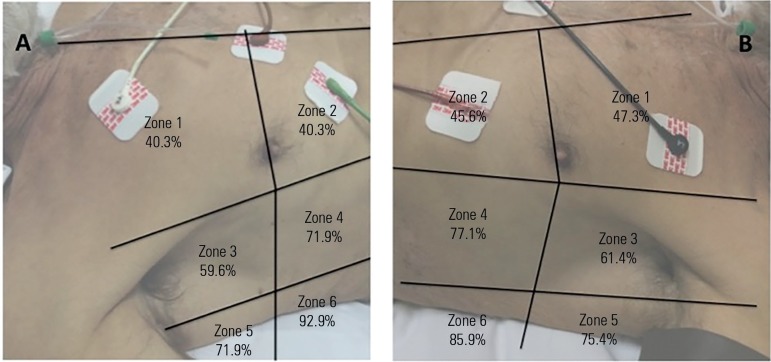
Prevalence of B-pattern and consolidation (C-lines) in 12 zones before spontaneous breathing trial in all 57 individuals. At the beginning of the T-piece trial, B-pattern and/or C-lines were already found at the lower and posterior lung regions in more than half of the individuals and remained non-aerated at the end of the trial. A - right side; B - left side.

A Siemens Sonoline G50 ultrasound machine and a 3.5-MHz curved array probe were used for all examinations. Patients were scanned while in the supine position. Using a longitudinal view, each intercostal space of upper and lower parts of the anterior, lateral, and posterior regions of the left and right chest wall were carefully examined (Figure 1).

**Figure 2:**
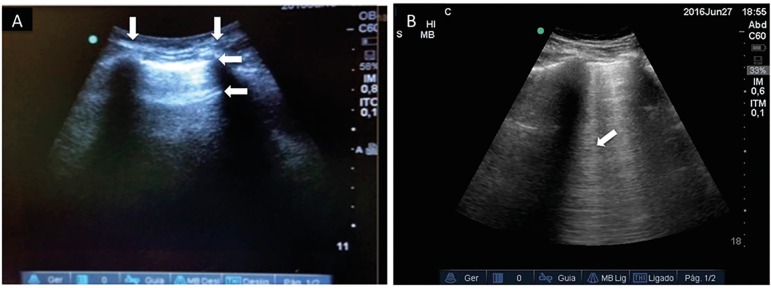
Lung ultrasound is largely based on the interpretation of artifacts created by the interplay of air and fluid in the lung. (A) The ribs on each side of the lung window (vertical arrows) form the bat wings of the "bat" sign, and the hyperechoic pleural line (horizontal arrow at the top) resemble the bat's body. Normal or well-aerated lung tissue leads to the formation of horizontal reverberation artifacts repeated in distance intervals roughly equal to the parietal pleura to the skin distance; these intervals are labeled A-lines (horizontal arrow below pleural line). (B) If the amount of fluid in the lung tissue is increased such as in pulmonary edema, the repetition artifacts multiply and vertical lines appear (B-lines - arrow), erasing A-lines.

The pleural line, sought between two rib shadows, indicates the pleural layers and generates a permanent landmark known as the bat sign. The pleural line displays sliding of the visceral pleura against the parietal pleura, a movement in rhythm with respiration. The normal lung surface associates lung sliding with horizontal repetitions of the pleural line, called A-lines. These lines indicate physiological or free gas (Figure 2A). B-lines are defined as discrete laser-like vertical hyperechoic reverberation artifacts that arise from the pleural line, extend to the bottom of the screen without fading, move synchronously with lung sliding, and erase A-lines. B-line reflects the coexistence of elements with a major acoustic impedance gradient, such as fluid and air. Fluid at the subpleural interlobular septum surrounded by air-filled alveoli fulfills this condition (Figure 2B).^(11,12,20,21)^ Three or more B lines in a single view are called a B-pattern. Presence of the B-pattern at two or more regions of the chest bilaterally characterizes interstitial edema.^(12)^ A C-line is a vertical line not originating at the pleural line but inside a consolidated lung tissue or on an irregular lung surface away from the pleural line, leading to ultrasound images similar to liver or splenic tissue. This line corresponds to non-aerated lung tissue such as seen in atelectasis, acute respiratory distress syndrome (ARDS) or pneumonia.^(22)^ In summary, from A to C-lines, there is a progressive decrease of the air-fluid ratio at the lung parenchyma.

Ultrasound evaluations were performed at the following time points: before starting SBT and at its conclusion - either after 30 - 120 minutes, prior to extubation or at the appearance of criteria for SBT interruption. The same trained investigator conducted the LUS assessment at each time point of the study. To avoid expeditious examinations in conditions of overwhelming respiratory distress, immediately before reconnecting the patient to the ventilator, we did not describe patterns of aeration other than the A-line, B-line and C-line; the number of single or confluent B-lines could not be reported.

At the beginning of the T-piece trial, B-pattern and/or C-lines were already found at the lower and posterior lung regions in more than half of the individuals and remained non-aerated at the end of the trial (Figure 1). According to the aforementioned papers,^(13,23)^ we postulated that a simplified approach on four chest anterior zones - 1 and 2 on the right and left sides - would be enough for the specific purpose of our study. This concept allowed a dichotomous approach to the lung. Therefore, despite collecting ultrasound data of 12 thoracic regions, only LUS findings on the following four anterior chest zones were analyzed: the intercostal space between the third and fourth ribs, and the intercostal space between the sixth and seventh ribs, to the left and right of the sternum and between the parasternal and midclavicular line. We noted B-predominance as any profile with an anterior bilateral B-pattern, based on previous studies.^(13,23)^

### Statistics

The results are expressed as the mean and standard deviation, median and interquartile range, and proportions, as appropriate. The normal distribution of the various parameters was investigated observing the distribution of data and the Shapiro-Wilk test. We used Fisher's exact test to compare proportions. Comparisons among the following three groups were made through one-way analysis of variance (ANOVA) for continuous variables with a normal distribution, and through the Kruskal-Wallis test for variables with a non-normal distribution: patients successfully extubated (successful SBT and extubation group); patients who failed the SBT (SBT failure group); andpatients reintubated within 48 hours (extubation failure group). The sensitivity, specificity, positive predictive value, negative predictive value, positive likelihood ratio and negative likelihood ratio of B-predominance for the prediction of SBT failure and extubation failure were calculated. A p value < 0.05 was considered statistically significant. Statistical analysis was performed with Statistical Package for Social Science (SPSS) version 20.0.

## RESULTS

All included individuals were successfully examined, and no dropouts due to poor examination conditions occurred. Forty-six subjects (80.7%) successfully completed the T-piece trial and were immediately extubated; 8 of these subjects required reintubation within 48 hours. The remaining 11 individuals had signs of poor tolerance during SBT and were reconnected to the ventilator. Overall, weaning failure (failed SBT and extubation) occurred in 19 patients (33%). Table 1 shows the baseline characteristics of the cohort according to outcomes. There was a higher prevalence of chronic obstructive pulmonary disease in the SBT failure group (54.5% *versus* 7.9% and 12.5% in the successful SBT and extubation group and extubation failure group, respectively). Sepsis from any source constituted the main reason for initiating MV in all groups. Thirty-four patients (59.6%) were extubated at the first attempt -, i.e., simple weaning patients.

**Table 1:** Characteristics of the study cohort

Patient characteristics (N = 57)	Successful SBT and extubation (N = 38)	SBT failure (N = 11)	Extubation failure (N = 8)	p value
Age (years)	70.6 (± 15.6)	70.9 (± 22.7)	82.7 (± 16.9)	0.17
Female Sex	16 (42.1)	6 (54.5)	3 (37.5)	0.72
APACHE II (points)	20 ± 6.8	22.6 ± 8.8	22.3 ± 4.4	0.47
SOFA score (points)	5.5 ± 2.9	7.6 ± 5.7	6.5 ± 4.4	0.26
BMI (kg/m^2^)	26.9 ± 5.6	23.7 ± 2.7	25.4 ± 7	0.26
RSBI (f/VT)	61.4 ± 21.73	71.1 ± 17.1	53 ± 17.8	0.44
MV duration (days)	5 (3 - 8.2)	7 (4 - 13)	5.5 (2.2 - 15.2)	0.50
48 hour-fluid balance prior to SBT (mL)	511.9 ± 3,080.45	1821.5 ± 2,720.29	747.50 ± 2,958.95	0.45
Co-morbidities				
COPD	3 (7.9)	6 (54.5)	1 (12.5)	0.04
Ejection fraction < 45%	3 (7.9)	2 (18.2)	0 (0)	0.37
LV diastolic dysfunction	11 (61.1)	2 (50)	6 (100)	0.18
Ischemic coronary disease	8 (21.1)	0 (0)	3 (37.5)	0.91
Renal replacement therapy	9 (23.7)	3 (27.3)	2 (25)	1.00
Ascitis	2 (5.3)	2 (18.2)	0 (0)	0.25
Reason for mechanical ventilation				
Respiratory sepsis	5 (13.2)	5 (45.5)	1 (12.5)	0.06
Non respiratory sepsis	14 (36.8)	1 (9.1)	1 (12.5)	0.13
Congestive heart failure	6 (15.8)	0 (0)	2 (25)	0.21
Coma	8 (21.1)	1 (9.1)	2 (25)	0.69
Postoperative ARF	1 (2.6)	0 (0)	0 (0)	1.00
COPD/Asthma	0 (0)	0 (0)	1 (12.5)	0.15
Pulmonary embolism	1 (2.6)	0 (0)	0 (0)	1.00
ARDS	2 (5.3)	2 (18.2)	0 (0)	0.25
Simple weaning	30 (78.9)	9 (81.8)	4 (50)	0.17

SBT - spontaneous breathing trial; APACHE II - Acute Physiology and Chronic Health Evaluation II; SOFA - Sequential Organ Failure Assessment; BMI - body mass index; RSBI - rapid shallow breathing index; MV - mechanical ventilation; COPD - chronic obstructive pulmonary disease; LV - left ventricular; ARF - acute respiratory failure; ARDS - acute respiratory distress syndrome. Data are presented as median (interquartile range), mean ± standard deviation or n (%).

In the SBT failure group, there was a slightly statistical trend of increasing B-predominance during the T-piece trial (p = 0.07). These subjects also exhibited higher B-predominance compared to the other groups at the end of trial (90% *versus* to 42.1% and 62.5% in the successful SBT and extubation and in extubation failure groups, respectively; p = 0.01). Although the difference did not reach significance (p = 0.26), the successful SBT and extubation group started the procedure with a lower B-predominance (39.5% compared to 63.6% and 50% in, respectively, the SBT failure and in extubation failure groups) (Table 2).

**Table 2:** B-predominance prior to spontaneous breathing trial and at the end of trial according to weaning groups

B-predominance	Successful SBT and extubation (n = 38)	SBT failure (n = 11)	Extubation failure (n = 8)	p value*
Before SBT	15 (39.5)	7 (63.6)	4 (50)	0.36
After SBT	16 (42.1)	9 (90)	5 (62.5)	0.01
p value†	0.4	0.07	0.27	

SBT - spontaneous breathing trial;

* For comparison among weaning groups at each moment;

† for comparison between before SBT and after SBT. Data are presented as n (%).

Table 3 shows the sensitivity, specificity, positive predictive value, negative predictive value, positive likelihood ratio and negative likelihood ratio of B-predominance for the prediction of SBT failure and extubation failure outcomes.

**Table 3:** Performance of B-predominance as a screening test for weaning prediction

Time of assessment	Outcome	Sensitivity	Specificity	PPV	NPV	PLR	NLR
Before SBT (n = 57)	SBT failure (n = 11)	0.64 (0.32 - 0.88)	0.59 (0.43 - 0.73)	0.27 (0.12 - 0.48)	0.87 (0.52 - 0.88)	1.54 (0.87 - 2.70)	0.62 (0.27 - 1.40)
Before SBT (n = 57)	SBT failure and extubation failure (n = 19)	0.58 (0.34 - 0.79)	0.60 (0.43 - 0.75)	0.42 (0.24 - 0.63)	0.74 (0.55 - 0.87)	1.47 (0.85 - 2.54)	0.69 (0.40 - 1.22)
After SBT (n = 46*)	Extubation Failure (n = 8)	0.62 (0.26 - 0.90)	0.58 (0.40 - 0.73)	0.24 (0.09 -	0.88 (0.68 - 0.97)	1.48 (0.77 - 2.85)	0.65 (0.26 - 1.64)

PPV - positive predictive value; NPV - negative predictive value; PLR - positive likelihood ratio; NLR - negative likelihood ratio; SBT - spontaneous breathing trial.

* Excluding failed SBT cases (not extubated). Data are expressed as estimated value (95% confidence interval).

## DISCUSSION

We presented an analysis of changes observed in LUS findings before and after SBT; while we acknowledge the low sample size of this study, our results lend credence to the idea that the increments of B-pattern on four anterior chest zones in subjects who failed T-piece trial represent a cardiac disturbance mechanism. Prior to conducting the T-piece trial, however, we were not able to identify individuals who would fail SBT or who would need reintubation within 48 hours.

Rapid changes in the respiratory and cardiac load occurring throughout SBT might manifest with dynamic changes in LUS that are only visible with real-time scanning. At the start of the trial, we could not demonstrate statistically significant differences in B-predominance among groups, conceivably because of type II error. During the trial, the SBT-failure group behaved differently, exhibiting higher increases in LUS B-predominance, similar to the other parameters of lung mechanics, hemodynamic performance and global tissue oxygenation.^(24)^ The clinical utility of such findings is uncertain because the clinical manifestations of severe respiratory distress were already evident at the moment of its detection.

The initiation of SBT after a period of MV is associated with some loss of lung aeration in critically ill subjects. Using the same LUS score technique as Bouhemad et al. (lower scores = better aeration),^(25)^ Soummer et al.^(26)^ showed that progressive lung derecruitment during an SBT identified patients likely to fail extubation. At the end of the SBT, patients with an LUS score of less than 13 had a 9% risk of post-extubation failure (4 of 43), whereas patients with an LUS score of more than 17 had an 85% risk of post-extubation failure (18 of 21). An end SBT LUS score between 13 and 17, seen in 25% of patients, did not allow for an accurate prediction of the extubation outcome. It may not be possible to draw conclusions regarding the risk of failed SBT prior to a T-piece trial.

Our data showed a lack of B-predominance accuracy to predict the need for reintubation within 48 hours. Given our small sample size, it is unclear whether considering the simplified four-region LUS protocol is truly imprecise for such purposes. However, considering that extubation failure might occur due to causes other than imbalances between cardiorespiratory capacity and load (failure to maintain airway patency due to upper airway edema, excessive secretions, inadequate muscle strength, neurological impairment, etc.), the behavior of the LUS findings during SBT might not portend reintubation rates accurately.

The quantification of pulmonary over-hydration was not the main scope of our investigation; however, from a practical point of view, the B-pattern indicates an increase in extravascular lung water with an absolute sensitivity.^(27)^ An association between the absence of B-lines detected by LUS and a low level of wedge pressure (pulmonary artery occlusion pressure) has been reported; nonetheless, B-predominance is observed in a wide range of pulmonary artery occlusion pressure values, precluding firm conclusions for the need of fluid withdrawal.^(23)^ Other observational studies demonstrated a better specificity of the finding of B-pattern in detecting elevated EVLW by the trans-pulmonary thermodilution method (PiCCO system).^(15,16)^ Enghard et al.^(14)^ applied a four-region LUS protocol and found a good correlation with trans-pulmonary thermodilution measurements. Finally, Dres et al.^(10)^ reported a link between SBT-induced increases in EVLW and weaning failure of cardiac origin with a specificity of 100%.

The present study is practical, qualitative, and highly reproducible.^(13,23)^ Documenting, for instance, the lateral walls, cardiac function, volume of pleural effusion, and vein calipers could provide additional information but would undermine simplicity. In this preliminary approach, the authors did not focus on posterior changes because posterior B-lines might indicate gravitational changes. Reducing scanning to four anterior chest zones is aimed to facilitate the initial assessment of this subset of patients through a simple, rapid and easy-to-perform method. Within 1 minute of LUS examination, researchers were able to acquire valuable information regarding the diagnosis of lung edema. The LUS score as presented^(25,26)^ has utility as a research tool, but might be overly complicated for the frontline intensivist to use in a busy ICU. We did not compare different protocols using, for example, an 8-, 12- or even a 28-zone approach, so no final conclusions could be drawn regarding the superiority of these approaches.

Our major limitations are the fact that this study was done at a single center and using a small sample size. Lung ultrasound examinations were performed only during working hours. The choice of a convenient sample and the small sample size also limit the interpretation and generalization of the findings. The overall rate of weaning failure was relatively high (33%). The rate of reintubation following extubation (17.4%) was, however, comparable to rates that have been reported before,^(28)^ as well as the prevalence of simple-weaning (75%),^(1-3)^ indicating that our prospective convenience sample had the same expected pre-test probability of SBT failure as any ordinary, medical-surgical ICU population. Like all techniques of ultrasonography, bedside LUS could be operator-dependent; however, a high intra- and inter-observer reproducibility has been reported.^(25)^

## CONCLUSION

Our study does not allow for general conclusions, but some important points could be inferred. Scanning of four regions is quite feasible and time-saving, as long as inferior and posterior B-lines might reflect gravitational changes. We speculate that a higher loss of lung aeration during a spontaneous breathing trial suggests weaning-induced cardiovascular dysfunction and increases in extravascular lung water.

The observation that spontaneous breathing trial-failure subjects display more severely deranged lung mechanics than successfully extubated subjects raises the question of whether these derangements might be detectable while patients are receiving full ventilatory support. Usual practice, physiology and well-known causes of weaning failure all support the use of lung ultrasound to identify patients who are at high risk of a failed spontaneous breathing trial. However, we do concede that these data need to be confirmed with an enlarged sample population to reduce the considerable data dispersion affecting the study. Therefore, we designed a multicenter observational study to evaluate whether lung ultrasound findings prior to T-piece trial are able to predict the earliest time that an individual might resume spontaneous breathing.
